# Supplementary material for: Risk Due to ABO Incompatibility and Donor-Recipient Weight Mismatch in Living Donor Kidney Transplantation: A National Cohort Study
Source: J Clin Med. 2021 Dec 1;10(23):5674. doi: 10.3390/jcm10235674 (PMC8658727; doi:10.3390/jcm10235674)
Supplement: Supplementary file 1 [file jcm-10-05674-s001.zip › jcm-1473413-supplementary.pdf]

Figure S1. Distribution of donor-recipient weight ratio

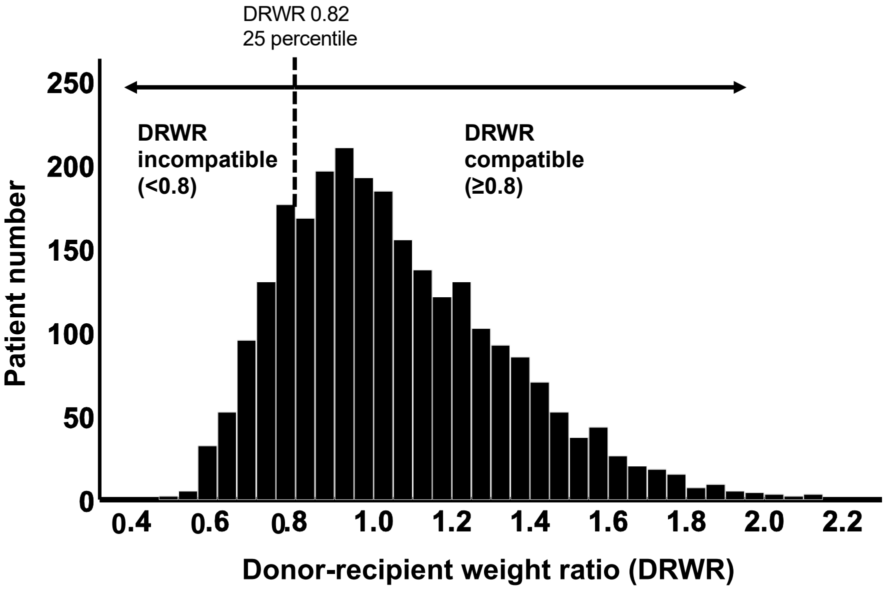

DRWR, dono-recipient weight ratio

Table S1. Details of ABOi LDKT (for available data only)

Variables	Available data	ABOi LDKT (n= 643)
Recipient blood type O	n=643	267 (41.5)
ABO titer, baseline		
IgM	n=249	32 (IQR 8-64)
IgG	n=175	32 (IQR 8-128)
High titer (≥ 256)		
ABO titer, at transplantation		
IgM	n=160	2 (IQR 1-4)
IgG	n=120	8 (IQR 2-16)
Rituximab use	n=306	290 (94.8)
Number of plasmapheresis	n=306	3.4±2.6
Intravenous immunoglobulin use	n=306	98 (32.0)

LDKT, living donor kidney transplantation

Table S2. Full results of one-way ANOVA with post hoc analyses for comparison eGFR at each time point

**eGFR at discharge**		***P* value**
ABOc-DRWRc (n=1570) 75.4±21.9	ABOc-DRWRi	<0.001
	ABOi-DRWRc	0.999
	ABOi-DRWRi	<0.001
ABOc-DRWRi (n=371) 59.1±15.6	ABOc-DRWRc	<0.001
	ABOi-DRWRc	<0.001
	ABOi-DRWRi	0.999
ABOi-DRWRc (n=519) 75.2±23.2	ABOc-DRWRc	0.999
	ABOc-DRWRi	<0.001
	ABOi-DRWRi	<0.001
ABOi-DRWRi (n=124) 57.8±16.5	ABOc-DRWRc	<0.001
	ABOc-DRWRi	0.999
	ABOi-DRWRc	<0.001
**eGFR at 6 months**		
ABOc-DRWRc (n=1542) 64.3±17.5	ABOc-DRWRi	<0.001
	ABOi-DRWRc	0.168
	ABOi-DRWRi	<0.001
ABOc-DRWRi (n=360) 57.1±14.7	ABOc-DRWRc	<0.001
	ABOi-DRWRc	<0.001
	ABOi-DRWRi	0.547
ABOi-DRWRc (n=513) 62.4±17.0	ABOc-DRWRc	0.168
	ABOc-DRWRi	<0.001
	ABOi-DRWRi	<0.001
ABOi-DRWRi (n=119) 54.1±16.9	ABOc-DRWRc	<0.001
	ABOc-DRWRi	0.547
	ABOi-DRWRc	<0.001
**eGFR at 1 year**		
ABOc-DRWRc (n=1435) 65.4±18.2	ABOc-DRWRi	<0.001
	ABOi-DRWRc	0.135
	ABOi-DRWRi	<0.001
ABOc-DRWRi (n=333) 58.0±16.2	ABOc-DRWRc	<0.001
	ABOi-DRWRc	<0.001
	ABOi-DRWRi	<0.546
ABOi-DRWRc (n=471) 63.2±18.7	ABOc-DRWRc	0.135
	ABOc-DRWRi	<0.001
	ABOi-DRWRi	<0.001
ABOi-DRWRi (n=111) 54.6±18.3	ABOc-DRWRc	<0.001
	ABOc-DRWRi	0.546
	ABOi-DRWRc	<0.001
**eGFR at 2 year**		
ABOc-DRWRc (n=1017) 65.0±19.6	ABOc-DRWRi	<0.001
	ABOi-DRWRc	0.444

	ABOi-DRWRi	<0.001
ABOc-DRWRi (n=237)<br>57.8±18.4	ABOc-DRWRc	<0.001
	ABOi-DRWRc	0.017
	ABOi-DRWRi	0.999
ABOi-DRWRc (n=325)<br>62.8±19.6	ABOc-DRWRc	0.444
	ABOc-DRWRi	0.017
	ABOi-DRWRi	0.003
ABOi-DRWRi (n=81)<br>54.4±20.8	ABOc-DRWRc	<0.001
	ABOc-DRWRi	0.999
	ABOi-DRWRc	0.003

DRWR, donor-recipient weight ratio

Post hoc analyses were performed with Bonferroni method.

Figure S2. Causes of graft failure in each group

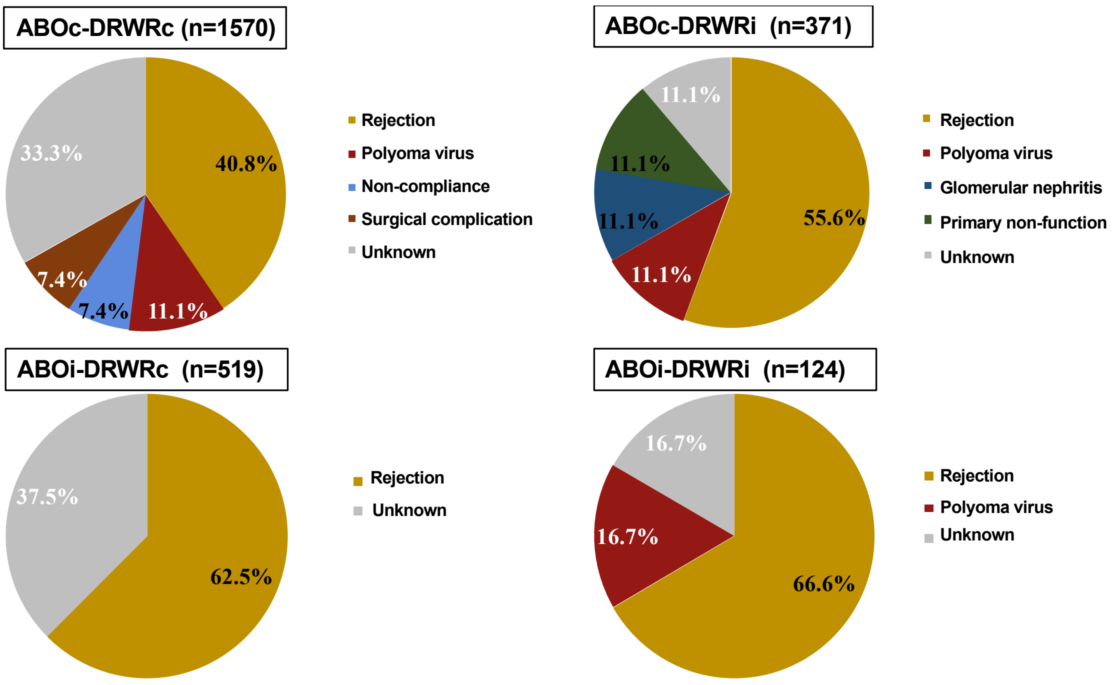

Table S3. Cox regression analysis for death-censored graft survival

Variables	Cox regression with competing risk analysis			
	Univariate		Multivariate	
	HR (95% CI)	p-value	HR (95% CI)	p-value
Age, per 1 year	0.98 (0.96-1.01)	0.079	0.97 (0.95-0.99)	0.026
Sex, male	1.37 (0.76-2.49)	0.294		
BMI per 1 kg/m^2	1.06 (0.98-1.13)	0.141		
Dialysis duration, month	1.00 (0.99-1.01)	0.361		
Retransplantation	0.32 (0.04-2.33)	0.261		
Diabetes mellitus	1.54 (0.87-2.70)	0.137		
Hypertension	0.58 (0.27-1.24)	0.162		
Cardiovascular disease	0.66 (0.21-2.12)	0.484		
Donor age	1.02 (1.00-1.05)	0.072	1.02 (0.99-1.05)	0.109
Donor sex, male	0.61 (0.34-1.11)	0.103		
Donor BMI	0.99 (0.90-1.08)	0.787		
Unrelated donor	1.33 (0.76-2.31)	0.318		
Number of HLA mismatches (for A/B/DR)	1.25 (1.02-1.52)	0.028	1.29 (1.04-1.60)	0.021
XM-DSA+	1.07 (0.33-3.45)	0.906		
Induction agent				
IL-2 receptor antibody	Reference			
Anti-thymocyte globulin	1.15 (0.49-2.70)	0.750		
Group				
ABOc DRWRc	Reference		Reference	
ABOc DRWRi	1.46 (0.69-3.10)	0.327	1.32 (0.62-2.83)	0.469
ABOi DRWRc	0.90 (0.41-1.98)	0.791	0.82 (0.37-1.81)	0.624
ABOi DRWRi	2.94 (1.21-7.12)	0.017	2.73 (1.11-6.70)	0.028
ABOi vs. ABOc	1.18 (0.64-2.19)	0.601	1.10 (0.59-2.04)	0.770
DRWRi vs. DRWRc	1.87 (1.02-3.43)	0.042	1.75 (0.95-3.23)	0.074

BMI, body mass index; DRWR, donor-recipient weight ratio; DSA, donor specific antibody;

HLA, human leukocyte antigen; IL, interleukin; XM, crossmatch

Figure S3. Causes of death in each group

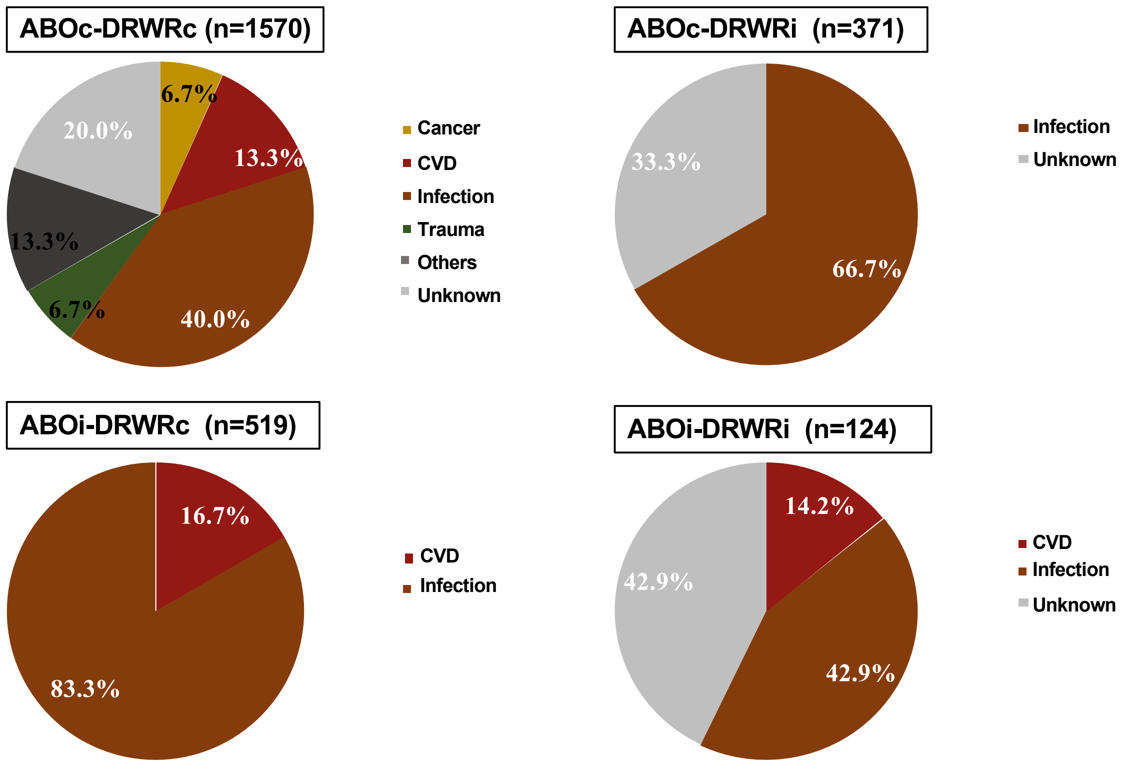

CVD, cardiovascular disease

Table S4. Cox regression analysis for patient survival

Variables	Cox regression with competing risk analysis			
	Univariate		Multivariate	
	HR (95% CI)	p-value	HR (95% CI)	p-value
Age	1.05 (1.01-1.08)	0.008		
Sex, male	2.19 (0.95-5.09)	0.067		
BMI	1.07 (0.98-1.17)	0.159		
Dialysis duration	1.01 (1.00-1.01)	0.093		
Retransplantation	1.09 (0.26-4.57)	0.907		
Diabetes mellitus	1.88 (0.93-3.81)	0.081		
Hypertension	3.36 (0.46-24.61)	0.234		
Cardiovascular disease	3.55 (1.59-7.95)	0.002	3.06 (1.35-6.91)	0.007
Donor age	1.01 (0.98-1.05)	0.418		
Donor sex, male	0.92 (0.45-1.87)	0.817		
Donor BMI	0.97 (0.86-1.08)	0.564		
Unrelated donor	4.16 (1.79-9.67)	0.001	3.32 (1.39-7.97)	0.007
Number of HLA mismatches (for A/B/DR)	1.55 (1.18-2.03)	0.002		
XM-DSA+	3.24 (1.24-8.44)	0.016	1.43 (1.04-1.98)	0.029
Induction agent				
IL-2 receptor antibody	Reference			
Anti-thymocyte globulin	2.01 (0.82-4.90)	0.125		
Group				
ABOc DRWRc	Reference		Reference	
ABOc DRWRi	0.86 (0.25-2.97)	0.813	0.67 (0.19-2.35)	0.533
ABOi DRWRc	1.21 (0.47-3.12)	0.693	0.93 (0.35-2.42)	0.875
ABOi DRWRi	6.02 (2.46-14.77)	<0.001	3.55 (1.39-9.04)	0.008
ABOi vs. ABOc	2.18 (1.07-4.45)	0.032	1.65 (0.79-3.42)	0.181
DRWRi vs. DRWRc	2.05 (0.96-4.34)	0.063	1.64 (0.75-3.59)	0.214

Multivariable Cox model for patient survival was established by stepwise regression for establishing optimal model with limited number of death event (n=30). CVD, unrelated donor and XM-DSA+ were finally included in the model.

BMI, body mass index; DRWR, donor-recipient weight ratio; DSA, donor specific antibody; HLA, human leukocyte antigen; IL, interleukin; XM, crossmatch

;

Table S5. Cox regression analysis for BPAR

Variables	Cox regression with competing risk analysis			
	Univariate		Multivariate	
	HR (95% CI)	p-value	HR (95% CI)	p-value
Age	0.99 (0.98-0.99)	0.001	0.98 (0.97-0.99)	<0.001
Sex, male	1.47 (1.22-1.77)	<0.001	1.59 (1.29-1.96)	<0.001
BMI	0.99 (0.98-1.02)	0.819		
Dialysis duration	0.99 (0.99-1.01)	0.179		
Retransplantation	0.53 (0.33-0.85)	0.008	0.56 (0.65-0.90)	0.016
Diabetes mellitus	0.91 (0.76-1.10)	0.336		
Hypertension	0.90 (0.69-1.18)	0.439		
Cardiovascular disease	0.72 (0.52-1.01)	0.055	0.73 (0.52-1.03)	0.073
Donor age	1.02 (1.01-1.03)	<0.001	1.02 (1.01-1.03)	<0.001
Donor sex, male	0.78 (0.65-0.93)	0.005	0.99 (0.82-1.21)	0.971
Donor BMI	1.01 (1.00-1.01)	0.083	1.01 (0.99-1.04)	0.083
Unrelated donor	1.37 (1.16-1.63)	<0.001	1.12 (0.89-1.40)	0.349
Number of HLA mismatches (for A/B/DR)	1.16 (1.09-1.23)	<0.001	1.14 (1.06-1.23)	0.001
XM-DSA+	1.15 (1.03-1.27)	0.010	1.21 (1.09-1.35)	<0.001
Induction agent				
IL-2 receptor antibody	Reference			
Anti-thymocyte globulin	1.44 (1.13-1.83)	0.003	1.29 (0.97-1.68)	0.063
Group				
ABOc DRWRc	Reference			
ABOc DRWRi	1.29 (1.02-1.64)	0.037	1.02 (0.79-1.32)	0.879
ABOi DRWRc	1.23 (0.99-1.52)	0.060	1.11 (0.89-1.38)	0.355
ABOi DRWRi	1.66 (1.17-2.34)	0.004	1.27 (0.88-1.82)	0.203
ABOi vs. ABOc	1.24 (1.03-1.50)	0.024	1.14 (0.94-1.38)	0.190
DRWRi vs. DRWRc	1.31 (1.07-1.60)	0.009	1.05 (0.84-1.31)	0.661

BMI, body mass index; DRWR, donor-recipient weight ratio; DSA, donor specific antibody;

HLA, human leukocyte antigen; IL, interleukin; XM, crossmatch

Table S6. Cox regression analysis for pneumonia

Variables	Cox regression with competing risk analysis			
	Univariate		Multivariate	
	HR (95% CI)	p-value	HR (95% CI)	p-value
Age	1.03 (1.01-1.04)	0.001	1.02 (1.01-1.04)	0.006
Sex, male	1.63 (1.12-2.38)	0.010	1.43 (0.96-2.14)	0.080
BMI	0.99 (0.95-1.03)	0.546		
Dialysis duration	1.00 (0.99-1.01)	0.706		
Retransplantation	0.88 (0.41-1.87)	0.732		
Diabetes mellitus	0.99 (0.69-1.44)	0.979		
Hypertension	1.25 (0.67-2.32)	0.479		
Cardiovascular disease	1.69 (1.04-2.75)	0.034	1.29 (0.78-2.13)	0.330
Donor age	1.02 (1.01-1.04)	0.003	1.02 (1.01-1.04)	0.006
Donor sex, male	0.83 (0.59-1.18)	0.304		
Donor BMI	0.98 (0.92-1.03)	0.386		
Unrelated donor	1.50 (1.07-2.11)	0.020	0.90 (0.61-1.33)	0.604
Number of HLA mismatches (for A/B/DR)	1.07 (0.95-1.20)	0.282		
XM-DSA+	0.97 (0.75-1.25)	0.804		
Induction agent				
IL-2 receptor antibody	Reference			
Anti-thymocyte globulin	0.99 (0.57-1.72)	0.964		
Group				
ABOc DRWRc	Reference			
ABOc DRWRi	1.34 (0.80-2.25)	0.262	1.15 (0.67-1.97)	0.605
ABOi DRWRc	1.91 (1.27-2.86)	0.002	1.87 (1.24-2.82)	0.003
ABOi DRWRi	3.53 (2.04-6.12)	<0.001	2.94 (1.64-5.57)	<0.001
ABOi vs. ABOc	2.08 (1.47-2.93)	<0.001	2.02 (1.42-2.87)	<0.001
DRWRi vs. DRWRc	1.53 (1.04-2.25)	0.030	1.28 (0.85-1.94)	0.235

BMI, body mass index; DRWR, donor-recipient weight ratio; DSA, donor specific antibody; HLA, human leukocyte antigen; IL, interleukin; XM, crossmatch
